# Pathogen Species Identification from Metagenomes in Ancient Remains: The Challenge of Identifying Human Pathogenic Species of *Trypanosomatidae* via Bioinformatic Tools

**DOI:** 10.3390/genes9080418

**Published:** 2018-08-20

**Authors:** Denis Sereno, Franck Dorkeld, Mohammad Akhoundi, Pascale Perrin

**Affiliations:** 1 IRD, Montpellier University, InterTryp, 34394 Montpellier, France; 2 INRA-UMR 1062 CBGP (INRA, IRD, CIRAD), Montpellier SupAgro, Montferrier-sur-Lez, 34988 Languedoc Roussillon, France; franck.dorkeld@inra.fr; 3 Parasitology-Mycology Department, Avicenne Hospital, AP-HP, 93000 Bobigny, France; m.akhoundi@yahoo.com; 4 Montpellier University, IRD, CNRS, MIVEGEC, 34394 Montpellier, France

**Keywords:** *Trypanosomatidae*, kraken taxonomic assignment tool, bowtie2 fast short reads aligner, ancient DNA, parasitome, co-infection

## Abstract

Accurate species identification from ancient DNA samples is a difficult task that would shed light on the evolutionary history of pathogenic microorganisms. The field of palaeomicrobiology has undoubtedly benefited from the advent of untargeted metagenomic approaches that use next-generation sequencing methodologies. Nevertheless, assigning ancient DNA at the species level is a challenging process. Recently, the gut microbiome analysis of three pre-Columbian Andean mummies (Santiago-Rodriguez et al., 2016) has called into question the identification of *Leishmania* in South America. The accurate assignment would be important because it will provide some key elements that are linked to the evolutionary scenario for visceral leishmaniasis agents in South America. Here, we recovered the metagenomic data filed in the metagenomics RAST server (MG-RAST) to identify the different members of the *Trypanosomatidae* family that have infected these ancient remains. For this purpose, we used the ultrafast metagenomic sequence classifier, based on an exact alignment of k-mers (Kraken) and Bowtie2, an ultrafast and memory-efficient tool for aligning sequencing reads to long reference sequences. The analyses, which have been conducted on the most exhaustive genomic database possible on *Trypanosomatidae*, show that species assignments could be biased by a lack of some genomic sequences of *Trypanosomatidae* species (strains). Nevertheless, our work raises the issue of possible co-infections by multiple members of the *Trypanosomatidae* family in these three pre-Columbian mummies. In the three mummies, we show the presence of DNA that is reminiscent of a probable co-infection with *Leptomonas seymouri*, a parasite of insect’s gut, and *Lotmaria*.

## 1. Introduction

Santiago-Rodriguez et al. reported for the first time some evidence of the occurrence of *Leishmania* DNA in the guts of Andean mummies dating to pre-Columbian times, and they proposed an assignment to *L. donovani* [1]. In South America, the circulation of such *Leishmania* species is currently unknown, and has never before been documented in human remains. Therefore, the proper identification of the *Leishmania* species that would have infected mummies before Iberian colonization remains a major concern, and would bring new elements to the puzzle of the possible evolutionary scenarios [2]. *L. donovani* and *L. infantum* are by far the most common *Leishmania* species responsible for the visceral form (visceral leishmaniasis, VL) of the disease in both the Old World and the New World. So far, all of the cases of leishmaniasis described on pre-Columbian mummies are reminiscent of cutaneous (CL) or mucocutaneous (MCL) lesions: CL was observed on a mummy dating to a cultural group from 700–800 AD and found in a cemetery in Peru [3], and MCL was found on four samples in the archaeological cemetery of Coyo Oriente (skulls approximately 1000 years old) located in the desert of San Pedro de Atacama, Northern Chile [4]. These sites come from a time period predating European contacts. A *Leishmania* infection was confirmed in these remains using a Polymerase chain reaction (PCR) approach [4] (amplification of fragments of the inositol monophosphate dehydrogenase gene, the kinetoplast minicircle, the amino acid permease AAP13LD, and the adenylate kinase gene). Furthermore, the amplified sequences differed from those of *L. donovani*.

The rise of NGS (next-generation sequencing) technologies has opened a new field of systematic investigations in metagenomics. When these new technologies are applied to samples of ancient human remains, they provide valuable information to scientists working on the evolutionary history of infectious diseases. To avoid environmental contamination and accurately authenticate ancient DNA, a number of precautions and rules have been enacted [5,6]. Among them: (i) the requirement of a dedicated ancient DNA laboratory to minimize and manage contamination, particularly during sample collection, and the risk of exogenous DNA contaminations from the laboratory; and (ii) microbial ancient DNA damage should exhibit patterns of DNA damage and fragmentation; nevertheless, these patterns vary according to source context and species, but also according to the workflow and enzymes used during library preparation. The characterization of gut microbiomes of pre-Columbian mummies by Santiago-Rodriguez et al. using unbiased metagenomic approaches gave the opportunity to detect traces of specific pathogens [1]. Nevertheless, several issues have been raised regarding the age of sequenced DNA and the possibility of environmental contaminations [7,8]. The answer to these questions has been published [9]. However, if environmental contamination is a crucial problem when working on bacteria, for human pathogenic *Trypanosomatidae*, environmental contamination is unlikely to occur. Indeed, these parasitic eukaryotes require an adequate environment to survive and proliferate. They are not free-living microorganisms, and have not had the ability to survive very long outside their hosts. Thus, contamination with such organisms is unlikely to occur during manipulation and the laboratory processing of samples. DNA contamination of the mummies by contact with some infected insects that transmit them (sandfly, *Psychodidae*-*Phlebotominae*, for *Leishmania* or *Triatoma*, *Reduviidae*-*Triatominae*, for *Trypanosoma cruzi)* cannot be ruled out. Thus, because of the very low probability of such contamination, the available metagenomic data [1] are of great interest to detect the presence of parasites belonging to the *Trypanosomatidae* familly in these ancient remains.

Basic Local Alignment Search Tool (BLAST) alignments, which rely on finding the best alignment to a panel of genomic sequences, were often the traditional approach to assign a taxonomic label to an unknown sequence. However, unambiguous assignment at the species level is very hard, and this tool is very expensive in central processing unit (CPU) time for NGS data analysis on local computers, even if new facilities are now available via the use of Cloud public infrastructure or computer cluster. To shed light on the causative agent of leishmaniasis infection (and more broadly on parasites belonging to the *Trypanosomatidae* family) in these ancient remains, we used new software dedicated to metagenomic data analysis. Kraken, a bioinformatic program [10], presents numerous advantages over other programs, including its speed of performing analysis on metagenomes. The identification at the species level by Kraken is based on the use of exact-match database queries of k-mers, rather than on alignment similarity. This new approach was applied to the metagenomic rapid annotation using subsystems technology (MG-RAST) pre-processed metagenomic data [1], and the results were compared to those obtained with Bowtie2 [11].

## 2. Materials and Methods

### 2.1. Data

The metagenomic data that we used of the gut microbiomes from three pre-Columbian Andean mummies (FI3, FI9, and FI12) were available in the MG-RAST server (http://blog.mg-rast.org/) (MGRAST IDs 4629033.3, 4630170.3 and 4626489.3, respectively).

We built a reference genome database composed of all of the complete *Trypanosomatidae* genomes collected from the NCBI (http://www.ncbi.nlm.nih.gov/genome/) and complemented by genomes from TriTrypDB release 37 (25 April 2018) (Kinetoplastid Genomics Resource http://tritrypdb.org/tritrypdb/) [12]. The total number of *Trypanosomatidae* genomes in the database is 79 (Table S1).

### 2.2. Methods

We analyzed the data with Kraken (version 1.0), which is a system for ultrafast metagenomic sequence classification using exact alignment [10] (http://ccb.jhu.edu/software/kraken/). It relies on the development of a database that contains records consisting of a k-mer and the lowest common ancestor (LCA) of all of the organisms whose genomes contain that k-mer [10]. We built a reference database with the default parameters of Kraken (k = 31). We downloaded the pre-processed sequences (this step filters sequences based on length, the number of ambiguous bases, and quality value) from the MG-RAST server, and then compared them with the non-redundant, custom-built *Trypanosomatidae* database. The results were visualized with a metagenomic visualization tool, Krona [13]. To complete the analysis, the same set of metagenome data was analyzed with Bowtie2—a program for the rapid alignment of gapped reads—using the sensitive option [11]. To extract the coverage from the Binary Alignment Map (BAM) alignment files, we used the samtools program version 1.08, command idxstats [14]. The reference genome database was used for analyses conducted with the two methodologies.

## 3. Results and Discussion

In a first attempt to compare the respective limits of the two tools in the species assignment, we focused on microorganisms belonging to the *Trypanosomatidae* family. These were chosen for two reasons: first, because reported *Leishmania* infection is already reported in some mummies [1], and second, because of the endemicity of Chagas disease [15] and leishmaniasis in this South American region [16].

We conducted analyses for the three mummies on pre-processed data, as indicated in the methods section. To confirm that the detected traces of *Trypanosomatidae* DNA are not the result of environmental contaminations, we used the data passed through the screening step (i.e., following the dereplication and the duplicate read inferred sequencing error estimation (DRISEE) steps) in MG-RAST. The results confirmed clearly a co-infection with at least two pathogenic Trypanosomatids and the fact that is not the result of a contamination

### 3.1. Trypanosomes and Chagas Disease

**Figure 1:**
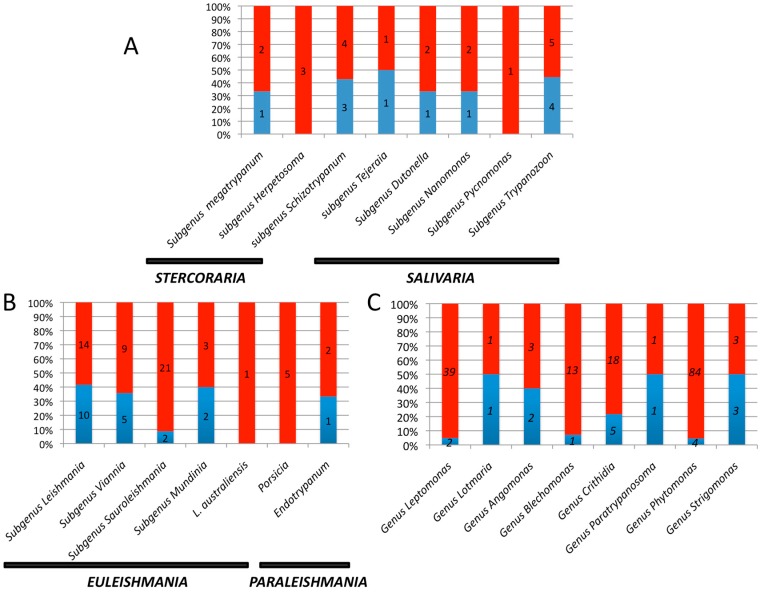
Proportion of available genomes (in blue) on the number of currently named species (in red) for the various genera belonging to the *Trypanosomatidae* family. (**A**) genus *Trypanosoma*; (**B**) genus *Leishmania*; (**C**) other *Trypanosomatidae* genera.

The genome representativity of our database is 55% (11 genomes out of approximately 20 *Trypanosoma* species currently described) (Figure 1A). The reference database includes trypanosomes responsible for American trypanosomiasis (*T. cruzi*) and trypanosomes responsible for human African trypanosomiasis and animal trypanosomiasis.

**Figure 2:**
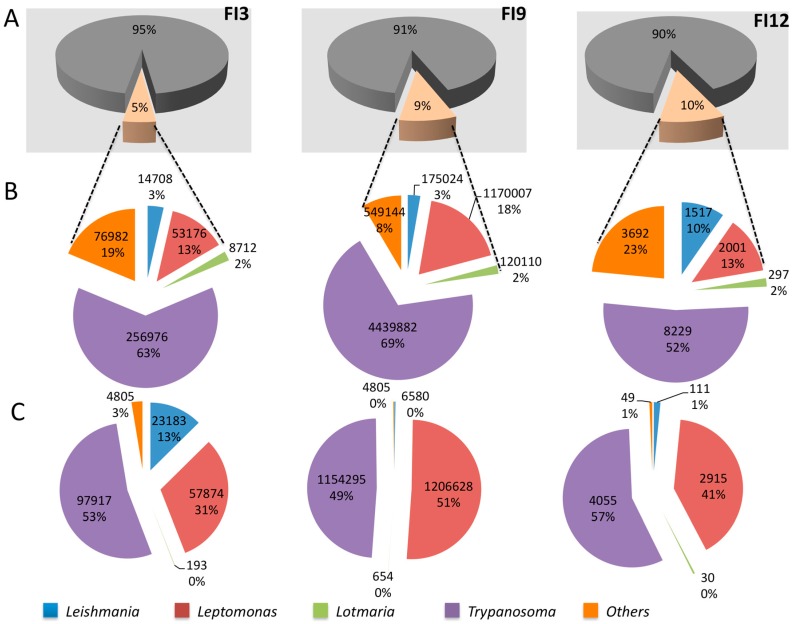
Composition of gut microbiota for the three Andean mummies studied by Santiago-Rodriguez et al. [1]: FI3, FI9, and FI12, respectively. (**A**) Proportion of reads assigned to the *Trypanosomatidae* family for each mummy after analysis with Kraken. Reads not matching *Trypanosomatidae* sequences appear in grey. Reads matching *Trypanosomatidae* sequences are flesh colored; (**B**) In *Trypanosomatidae*: numbers and percentages of reads assigned to the genera *Trypanosoma* (purple), *Leishmania* (blue), *Leptomonas* (red), *Lotmaria* (green). Reads from mummies FI3, FI9, and FI12 matching to any other *Trypanosomatidae* are shown in orange; (**C**) Results obtained by the Bowtie2 alignment sequence for mummies FI3, FI9, and FI12, respectively. For facility in comparison of the results gathered with both software the same color reference was used.

**Figure 3:**
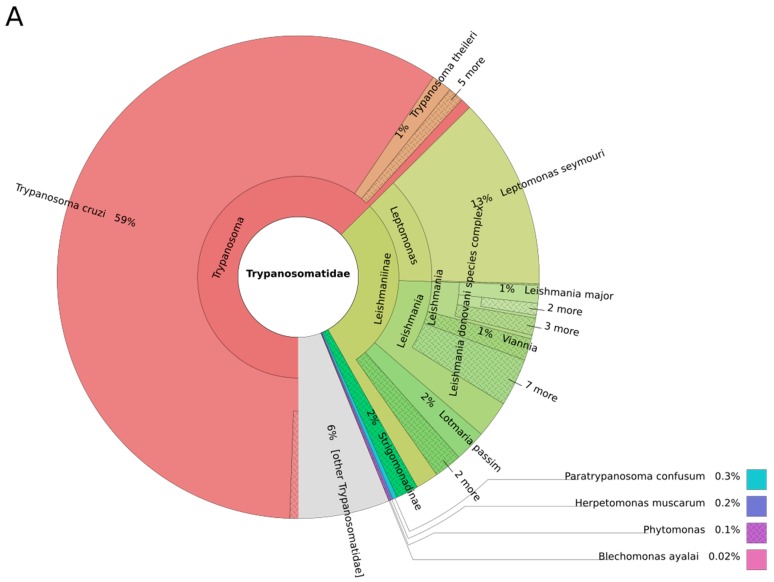

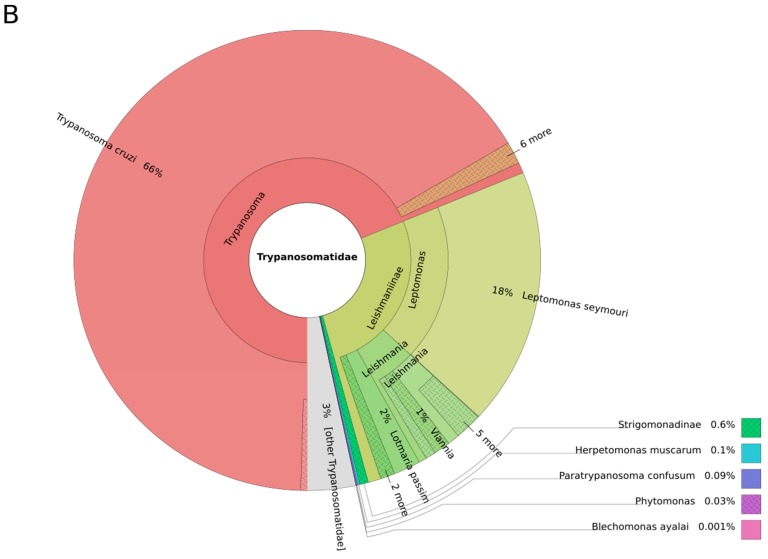

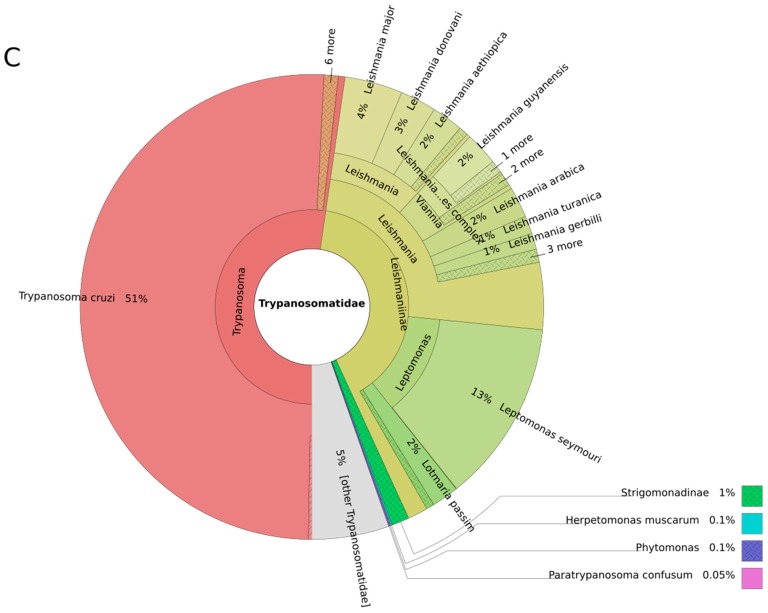
Profiles of microbiomes from three Andean mummies [1]: FI3, FI9, and FI12, for *Trypanosomatidae*. Circles represent taxonomic classification in ascending order up to the species level (outermost circle) with their relative abundance. These graphs were generated using the program Krona. Less abundant taxa are listed outside the charts.

The analysis performed with Kraken reveals that a non-negligible proportion of reads (5%, 9%, and 10% for FI3, FI9, and FI12) is attributed to the *Trypanosomatidae* family (Figure 2A). The genus *Trypanosoma* is clearly identified in ancient DNA from the guts of the three mummies FI3, FI9, and FI12, with 63%, 69%, and 52% of the detected *Trypanosomatidae* community, respectively (Figure 2B). As expected, almost all of the reads belonging to the genus *Trypanosoma* can be attributed to *T. cruzi* (Figure 3A–C) for the three mummies. These results corroborate the conclusion of Santiago-Rodriguez et al. [1]. Currently, *T. cruzi* is split into six genetic lineages or discrete typing units (DTUs) named TcI, TcIV, TcII, TcIII, TcV, and TcVI, respectively, and a seventh one called TcBat. For both FI3 and FI9, the highest number of reads matches the *T. cruzi* strain Tula cl2 (Table S2), which belongs to TcI (DTU I). This DTU is widely represented in the genomic database that we gathered (five of the 11 genomes currently available). For FI12, reads that match Tula cl2 are scarce (Table S2). Nevertheless, because of the relatively low proportion (<10%) of reads that match with a genome filed in our database, it is probable that the DTU of the infecting *T. cruzi* strain is not yet represented.

Analysis with Bowtie2 clearly confirmed a *T. cruzi* infection (53%, 49%, and 57% for FI3, FI9, and FI12, respectively) in these mummies (Figure 2C). The majority of reads matched *T. cruzi* strains Y and Tula cl2 (Table S3) rather than other genomes filed in the database. Concerning the alignment with *T. cruzi* Y, the reads matched 178 and 203 contigs in FI3 and FI9, respectively, out of 9821 (Table S4). A large majority of reads (80% in FI3 and 90% in FI9) matched 52 identical contigs for both mummies. For *T. cruzi* Tula cl2, reads of mummy FI3 and mummy FI9 matched 42 and 39 contigs, respectively, out of 5300, and almost 100% of the reads for both mummies matched 28 identical contigs (Table S4). Overall, this set of results indicates that the infecting *T. cruzi* strain is probably not in our reference database.

Most of the genomes of *T. cruzi* strains are lacking: currently, only 11 complete genomes are available in the NCBI and/or TriTrypDB databases out of more than 1902 deposited strains to date [17]. Likewise, the database we used contains no representative genomes belonging to DTUs III, IV, or VII (TcBat). Interestingly, ancient *T. cruzi* DNA has also been identified in human mummies dating from the same period (Chinchorro culture) and in the same geographical region, Southern Peru [18]. In Bolivia and Peru, strains belonging to the DTU I and DTU V clades are the main *T. cruzi* strains isolated, followed by strains of DTUs IV, III, and VI [15]. The knowledge of the complex evolutionary history of *T. cruzi*, which involved genetic exchanges [19], and the existence of hybrid DTUs will certainly benefit from the identification of the infecting strains at the DTU level from this ancient DNA material. Insight into the circulating strains in these mummies should generate important elements for the calibration of such a dynamically evolving scenario. Nevertheless, our analysis highlights that the ancient *T. cruzi* DNA present in the three mummies cannot be assigned to *T. cruzi* CL Brener (DTU VI).

### 3.2. Leishmania and Leishmaniasis

All of the *Leishmania* genomes that are currently available in NCBI or in TriTrypDB are included in the reference database. The database represents only 38% of genomes from 21 of the 54 *Leishmania* species that have been currently identified [20]. Microorganisms from the subgenera *Leishmania* and *Viannia* are well represented (Figure 1B). Human pathogenic *Leishmania* spp. are far better represented in the database (14/20), with the subgenera *Leishmania* and *Viannia* well represented (80% and 83%, respectively). No genome from the subgenus *Porcisia* (*Paraleishmania* section) is available (Table 1). This subgenus includes some human pathogenic *Leishmania* sp., such as *L. colombiensis*.

The NGS reads analyzed with the metagenomic sequence classifier Kraken highlight a probable co-infection with a *Leishmania* parasite for the three mummies FI3, FI9, and FI12 (Figure 2B), with 3%, 3%, and 10% of the reads attributed to *Trypanosomatidae* that belong to the genus *Leishmania*, respectively. From Figure 3, it is clear that Kraken assigns reads to a large number of *Leishmania* species, each with a low percentage. Clearly, no predominant *Leishmania* spp. is detected, and *L. donovani* does not appear as a probable infecting *Leishmania* species in all of these mummies. Therefore, these results prompt us to question the *Leishmania* species infecting the mummies. Since DNA originates from internal tissues, we have to look for the *Leishmania* spp. That are currently known to affect mainly internal organs and cause VL, namely, *L. donovani*, *L. infantum*, *L. tropica*, *L. martiniquensis*, and *L. colombiensis*. At this point, we can exclude an infection by the first four L. species cited above. The sequence of the *L. colombiensis* genome (*Paraleishmania* section) is currently not available, and may be the *Leishmania* agent that we are seeking. Currently, in Peru, the country from which mummies originate, *Leishmania* pathogens for humans include *L. peruviana*, *L. guyanensis*, *L. amazonensis*, *L. lainsoni*, and *L. braziliensis* [20]. Some *Leishmania* species that usually cause cutaneous forms have, under some circumstances, the capacity to disseminate into internal organs [21,22]. Such unusual clinical presentation is frequently associated with an immunocompromised state or dysfunction of the T helper-mediated immune response [2]. Our study clearly shows that a low proportion of reads are attributed to *L. peruviana*, *L. amazonensis*, *L. braziliensis*, and *L. guyanensis* (species causing CL). Such low frequency in read assignment calls into question the validity of the identification (Figure 2) (Table S2). Nevertheless, the occurrence of an infection by multiple *Leishmania* sp. cannot be ruled out as well as infection by hybrid strains of *Leishmania* [20]. From these results, it is clear that to be accurate in its species assignment, the reference database used by Kraken needs to be highly representative of the described species and strains. The more exhaustive the database, the better the assignment accuracy will be.

The alignment program Bowtie2 detects the presence of *Leishmania* in three mummies (FI3, FI9, and FI12) (Figure 2C). However, unlike the mummy FI12, in the mummies FI3 and FI9, the reads match a large panel of *Leishmania* sp., especially in the mummy FI3 (Table S3). Then, it is not possible to unequivocally assign DNA to a specific *Leishmania* species and *L. donovani* in particular due to the number of genomes of different *Leishmania* spp. matched.

Our analysis reveals that the genome of the infecting *Leishmania* species, whose DNA is detected in these ancient remains, is probably not present in our reference database. We may consider the occurrence of an ancient Leishmania species. Nevertheless, since our reference database is not exhaustive, we cannot rule out the presence of *Leishmania* sp. causing CL or MCL or the co-infection of multiple *Leishmania* spp. Still, the detection of *Leishmania* DNA is clearly not the result of an artefact or a contamination.

### 3.3. Other Leishmaniinae (Crithidiatae)

*Trypanosoma* and *Leishmania* are dixenous parasites, meaning that their life cycle includes an invertebrate as a first host and a vertebrate as a second host. Several genomes of monoxenous (one-host) *Trypanosomatidae* are available and belong to the genera *Angomonas*, *Crithidia*, *Leptomonas*, *Lotmaria,* and *Strigomonas* (Table S1). The database genome representativity for *Leptomonas* is 5%, with only two genomes available out of the 39 *Leptomonas* species currently described (Figure 1C). The analysis performed on the three mummies revealed that a relatively high proportion of reads attributed to the family *Trypanosomatidae* is reminiscent of the genus *Leptomonas* (13%, 18%, and 13% for F13, FI9, and FI12, respectively) (Figure 2B). A large majority of these reads are assigned to *L. seymouri* and not *L. pyrrochoris* after an analysis with Kraken (Figure 3A–C) (Table S2). Nevertheless, because of the lack of available genomes for 37 out of 39 *Leptomonas* sp., the assignment to *L. seymouri* has to be taken with caution, and an assignment at the strain level cannot be made.

Bowtie2 also confirms the recurrent occurrence of *L. seymouri* in all of the mummies (Figure 2C), and a high number of reads match the *L. seymouri* strain BHU-1095 (Table S3) in mummies FI3 and FI9 (56,245 and 125,577, respectively). In both mummies, the reads match with eight and nine scaffolds (out of 1216), respectively, and a large majority of reads match on only five scaffolds (Table S4). *Leptomonas* species are usually found in the gut of insects, but they have the potential to infect mammals as an opportunistic parasite. Nevertheless, their infective capacity in mammals seems to be limited to immunocompromised hosts [23]. Interestingly, *L. seymouri* has been repeatedly isolated from VL patients infected by *L. donovani* in India [24].

To our surprise, the analysis performed with Kraken reveals that an equivalent proportion of reads (2%) is assigned to *L. passim* in the three mummies (Figure 2B and Figure 3). Via Bowtie2 analysis, a very low number of reads matches this genus in the mummies (Figure 2C and Table S3). *L. passim* is the founding member of this genus (Figure 1). This member of Crithidiatae is described as a common parasite of the honey bee *Apis mellifera* [25], which calls into question its presence in a human gut microbiome.

## 4. Conclusions

The strength of our approach is to work with the most exhaustive reference database of *Trypanosomatidae* genomic sequences that are possible, combined with two programs that are complementary in their metagenomic analysis approaches (k-mer searching versus local alignment). These allowed us to gain insight into the identity of the infecting *Trypanosomatidae* agent. Analysis using the sequence classifier (Kraken) unambiguously confirmed *T. cruzi* infection and undoubtedly *Leishmania* infection in the three Andean mummies. In addition, our analysis provides new information on co-infection by at least two human pathogenic trypanosomatids, *Leishmania* spp. and *T. cruzi*, in all of the mummies with available metagenomes. This type of co-infection is known to occur in humans as well as in some wild mammals [26,27]. Unfortunately, it is not possible to go further in the assignment to the species level for *Leishmania* and DTU level for *T. cruzi*. This may be due to the lack of a number of *Leishmania* genomes, particularly those of the *Paraleishmania* section, and of members of *T. cruzi* belonging to some DTUs. It also highlights a pattern of polyinfection coupled with an opportunistic trypanosomatid, i.e., *L. seymouri* [28]. Therefore, future studies with an exhaustive reference database are necessary to better understand the interrelationships that shape the microbial community and play a role in the evolution of the parasitome.

## Figures and Tables

**Table 1:** Representativity of sequenced genomes available in relation to the number of known *Leishmania* species that are pathogenic for humans, according the updated classification of Akhoundi et al. (2017) [18].

	*Leishmania*	*Viannia*	*Sauroleishmania*	*Mundinia*	*L. australiensis* *(L. macropodum)*	*Porcisia*	*Endotrypanum*
Number of species pathogenic for humans	10	6	0	1	1	2	0
Number of genomes available	8	5	0	1	0	0	0
Representativity in %	80	83	0	100	0	0	0

## Supplementary Materials

The following are available online at http://www.mdpi.com/2073-4425/9/8/418/s1, Table S1: List of *Trypanosomatidae* genome sequences available and included in the reference database. Table S2. (A) Results of Kraken analysis on preprocessed metagenomic data for the mummy FI3 (mgm_46.29033.3); (B) results of Kraken analysis on preprocessed metagenomic data for the mummy FI9 (mgm_46.30170.3); (C) results of Kraken analysis on preprocessed metagenomic data for the mummy FI12 (mgm_46.26489.3); Table S3. Results of Bowtie2 analysis concerning the three mummies FI3, FI9 and FI12. Table S4. Distribution of reads matching (a) *Leptomonas seymouri*, (b) *Trypanosoma cruzi* Y and (c) *Trypanosoma cruzi* Tula cl2 on the contigs for the three mummies.
