# Supplementary material for: Pathogen Species Identification from Metagenomes in Ancient Remains: The Challenge of Identifying Human Pathogenic Species of Trypanosomatidae via Bioinformatic Tools
Source: Genes (Basel). 2018 Aug 20;9(8):418. doi: 10.3390/genes9080418 (PMC6115896; doi:10.3390/genes9080418)
Supplement: Supplementary file 1 [file genes-09-00418-s001.zip › SUPPLEMENTARY/Table S2_Published-version.docx]

**Table S2 : Results of KRAKEN analysis on preprocessed metagenomic data for the mummy A) FI3, B) FI9 and C) FI12**

**A ) Results of Kraken analysis on Trypanosomatidae for the mummy FI3 (mgm_46-29033.3)**

94.91 7654001 7654001 U 0 unclassified

5.09 410554 0 - 1 root

5.09 410554 0 - 131567 cellular organisms

5.09 410554 0 D 2759 Eukaryota

5.09 410554 0 - 33682 Euglenozoa

5.09 410554 0 O 5653 Kinetoplastida

5.09 410554 25192 F 5654 Trypanosomatidae

3.19 256976 2866 G 5690 Trypanosoma

3.03 244152 0 - 47570 Schizotrypanum

3.03 244152 201491 S 5693 Trypanosoma cruzi

0.37 29974 29974 - 1206070 Trypanosoma cruzi Tula cl2

0.09 7372 7372 - 366581 Trypanosoma cruzi strain Esmeraldo

0.03 2308 2308 - 85056 Trypanosoma cruzi marinkellei

0.02 1810 1810 - 914063 Trypanosoma cruzi JR cl. 4

0.01 1197 1197 - 1416333 Trypanosoma cruzi Dm28c

0.08 6271 93 - 669453 Trypanosoma with unspecified subgenus

0.07 5588 5588 S 67003 Trypanosoma theileri

0.01 590 590 S 71804 Trypanosoma grayi

0.03 2116 658 - 39700 Trypanozoon

0.01 1186 7 S 5691 Trypanosoma brucei

0.01 773 0 - 5702 Trypanosoma brucei brucei

0.01 773 773 - 185431 Trypanosoma brucei brucei TREU927

0.01 406 0 - 31285 Trypanosoma brucei gambiense

0.01 406 406 - 679716 Trypanosoma brucei gambiense DAL972

0.00 272 272 S 5694 Trypanosoma equiperdum

0.01 648 0 - 47571 Duttonella

0.01 648 0 S 5699 Trypanosoma vivax

0.01 648 648 - 1055687 Trypanosoma vivax Y486

0.01 641 0 - 47569 Nannomonas

0.01 641 518 S 5692 Trypanosoma congolense

0.00 123 123 - 1068625 Trypanosoma congolense IL3000

0.00 282 0 - 39701 Herpetosoma

0.00 282 0 S 5698 Trypanosoma rangeli

0.00 282 282 - 429131 Trypanosoma rangeli SC58

1.49 119820 6254 - 1286322 Leishmaniinae

0.66 53176 0 G 5683 Leptomonas

0.65 52766 52704 S 5684 Leptomonas seymouri

0.00 62 62 - 1263718 Leptomonas seymouri BHU1095

0.01 410 410 S 157538 Leptomonas pyrrhocoris

0.55 44042 8574 G 5658 Leishmania

0.18 14708 1001 - 38568 Leishmania

0.06 4914 0 - 38581 Leishmania major species complex

0.06 4914 3219 S 5664 Leishmania major

0.01 768 768 - 860570 Leishmania major strain SD 75.1

0.01 527 527 - 860569 Leishmania major strain LV39c5

0.00 400 400 - 347515 Leishmania major strain Friedlin

0.05 4032 753 - 38574 Leishmania donovani species complex

0.04 2925 1294 S 5661 Leishmania donovani

0.01 1094 1094 - 1215323 Leishmania donovani Ld 2001

0.01 537 537 - 1240127 Leishmania donovani Ld 39

0.00 354 0 S 5671 Leishmania infantum

0.00 354 354 - 435258 Leishmania infantum JPCM5

0.03 2718 107 - 38582 Leishmania mexicana species complex

0.01 1150 1150 S 5663 Leishmania enriettii

0.01 823 0 S 5665 Leishmania mexicana

0.01 823 823 - 929439 Leishmania mexicana MHOM/GT/2001/U1103

0.01 638 638 S 5659 Leishmania amazonensis

0.01 1166 0 - 38584 Leishmania tropica species complex

0.01 1166 0 S 5666 Leishmania tropica

0.01 1166 1166 - 1206058 Leishmania tropica L590

0.01 877 0 - 38570 Leishmania aethiopica species complex

0.01 877 0 S 5667 Leishmania aethiopica

0.01 877 877 - 1206056 Leishmania aethiopica L147

0.11 9153 1622 - 40283 unclassified Leishmania

0.04 3195 3195 S 40284 Leishmania arabica

0.04 3127 3127 S 62297 Leishmania turanica

0.01 985 985 S 40285 Leishmania gerbilli

0.00 123 123 S 1638716 Leishmania sp. AIIMS/LM/SS/PKDL/LD-974

0.00 101 101 S 1303197 Leishmania sp. MAR LEM2494

0.07 5984 387 - 37616 Viannia

0.03 2669 420 - 38579 Leishmania guyanensis species complex

0.02 1396 1396 S 5670 Leishmania guyanensis

0.01 853 317 S 5679 Leishmania panamensis

0.01 536 536 - 1295824 Leishmania panamensis MHOM/COL/81/L13

0.02 1570 111 - 37617 Leishmania braziliensis species complex

0.01 937 205 S 5660 Leishmania braziliensis

0.01 483 483 - 420245 Leishmania braziliensis MHOM/BR/75/M2904

0.00 249 249 - 1295825 Leishmania braziliensis MHOM/BR/75/M2903

0.01 522 522 S 5681 Leishmania peruviana

0.02 1358 0 - 38583 Leishmania naiffi species complex

0.02 1358 1358 S 5678 Leishmania naiffi

0.07 5623 21 - 5688 lizard Leishmania

0.04 3192 3192 S 5689 Leishmania tarentolae

0.03 2410 2410 S 5674 Leishmania adleri

0.11 8712 0 G 1620386 Lotmaria

0.11 8712 8712 S 1620387 Lotmaria passim

0.05 3971 122 G 5655 Crithidia

0.02 1570 1570 S 5656 Crithidia fasciculata

0.01 1148 1148 S 288676 Crithidia bombi

0.01 503 503 S 796354 Crithidia expoeki

0.00 321 321 S 796356 Crithidia mellificae

0.00 307 307 S 59798 Crithidia acanthocephali

0.05 3665 0 G 5703 Endotrypanum

0.05 3665 3665 S 5705 Endotrypanum monterogeii

0.08 6195 97 - 1581334 Strigomonadinae

0.05 4326 25 G 1003010 Strigomonas

0.02 1722 1722 S 1003336 Strigomonas galati

0.02 1657 1657 S 5657 Strigomonas oncopelti

0.01 922 922 S 28005 Strigomonas culicis

0.02 1772 4 G 1003337 Angomonas

0.02 1464 1464 S 59799 Angomonas deanei

0.00 304 304 S 59800 Angomonas desouzai

0.01 1169 0 G 1470208 Paratrypanosoma

0.01 1169 1169 S 1470209 Paratrypanosoma confusum

0.01 628 0 G 5714 Herpetomonas

0.01 628 628 S 5718 Herpetomonas muscarum

0.01 474 7 G 5706 Phytomonas

0.00 210 0 S 5707 Phytomonas serpens

0.00 210 210 - 1276585 Phytomonas serpens 9T

0.00 151 151 S 1902498 Phytomonas francai

0.00 106 0 - 470182 unclassified Phytomonas

0.00 98 98 S 134006 Phytomonas sp. isolate EM1

0.00 8 8 S 134013 Phytomonas sp. isolate Hart1

0.00 100 0 G 1463229 Blechomonas

0.00 100 100 S 1463230 Blechomonas ayalai

**B ) Results of Kraken analysis on Trypanosomatidae for the mummy FI9 (mgm_46-30170.3)**

90.79 63587893 63587893 U 0 unclassified

9.21 6454167 0 - 1 root

9.21 6454167 0 - 131567 cellular organisms

9.21 6454167 0 D 2759 Eukaryota

9.21 6454167 0 - 33682 Euglenozoa

9.21 6454167 0 O 5653 Kinetoplastida

9.21 6454167 216386 F 5654 Trypanosomatidae

6.34 4439882 47622 G 5690 Trypanosoma

6.13 4291456 0 - 47570 Schizotrypanum

6.13 4291456 3780754 S 5693 Trypanosoma cruzi

0.52 361260 361260 - 1206070 Trypanosoma cruzi Tula cl2

0.13 88914 88914 - 366581 Trypanosoma cruzi strain Esmeraldo

0.04 28818 28818 - 85056 Trypanosoma cruzi marinkellei

0.03 24115 24115 - 914063 Trypanosoma cruzi JR cl. 4

0.01 7595 7595 - 1416333 Trypanosoma cruzi Dm28c

0.07 50713 0 - 47571 Duttonella

0.07 50713 0 S 5699 Trypanosoma vivax

0.07 50713 50713 - 1055687 Trypanosoma vivax Y486

0.05 32743 36 - 669453 Trypanosoma with unspecified subgenus

0.04 30578 30578 S 67003 Trypanosoma theileri

0.00 2129 2129 S 71804 Trypanosoma grayi

0.02 14676 5339 - 39700 Trypanozoon

0.01 6586 365 S 5691 Trypanosoma brucei

0.01 3845 0 - 31285 Trypanosoma brucei gambiense

0.01 3845 3845 - 679716 Trypanosoma brucei gambiense DAL972

0.00 2376 0 - 5702 Trypanosoma brucei brucei

0.00 2376 2376 - 185431 Trypanosoma brucei brucei TREU927

0.00 2751 2751 S 5694 Trypanosoma equiperdum

0.00 1606 0 - 47569 Nannomonas

0.00 1606 398 S 5692 Trypanosoma congolense

0.00 1208 1208 - 1068625 Trypanosoma congolense IL3000

0.00 1066 0 - 39701 Herpetosoma

0.00 1066 0 S 5698 Trypanosoma rangeli

0.00 1066 1066 - 429131 Trypanosoma rangeli SC58

2.49 1741812 57040 - 1286322 Leishmaniinae

1.67 1170007 0 G 5683 Leptomonas

1.67 1167050 1166076 S 5684 Leptomonas seymouri

0.00 974 974 - 1263718 Leptomonas seymouri BHU1095

0.00 2957 2957 S 157538 Leptomonas pyrrhocoris

0.47 329624 33646 G 5658 Leishmania

0.25 175024 54400 - 38568 Leishmania

0.06 45458 3113 - 38582 Leishmania mexicana species complex

0.05 33096 0 S 5665 Leishmania mexicana

0.05 33096 33096 - 929439 Leishmania mexicana MHOM/GT/2001/U1103

0.01 6396 6396 S 5663 Leishmania enriettii

0.00 2853 2853 S 5659 Leishmania amazonensis

0.04 26066 0 - 38570 Leishmania aethiopica species complex

0.04 26066 0 S 5667 Leishmania aethiopica

0.04 26066 26066 - 1206056 Leishmania aethiopica L147

0.03 18381 359 - 38574 Leishmania donovani species complex

0.02 13476 3682 S 5661 Leishmania donovani

0.01 6541 6541 - 1215323 Leishmania donovani Ld 2001

0.00 3253 3253 - 1240127 Leishmania donovani Ld 39

0.01 4546 0 S 5671 Leishmania infantum

0.01 4546 4546 - 435258 Leishmania infantum JPCM5

0.03 18053 0 - 38581 Leishmania major species complex

0.03 18053 6528 S 5664 Leishmania major

0.01 5539 5539 - 860569 Leishmania major strain LV39c5

0.00 3283 3283 - 860570 Leishmania major strain SD 75.1

0.00 2703 2703 - 347515 Leishmania major strain Friedlin

0.02 12666 0 - 38584 Leishmania tropica species complex

0.02 12666 0 S 5666 Leishmania tropica

0.02 12666 12666 - 1206058 Leishmania tropica L590

0.10 73034 6995 - 37616 Viannia

0.06 43572 0 - 38583 Leishmania naiffi species complex

0.06 43572 43572 S 5678 Leishmania naiffi

0.02 11746 2282 - 37617 Leishmania braziliensis species complex

0.01 6141 187 S 5660 Leishmania braziliensis

0.00 2978 2978 - 420245 Leishmania braziliensis MHOM/BR/75/M2904

0.00 2976 2976 - 1295825 Leishmania braziliensis MHOM/BR/75/M2903

0.00 3323 3323 S 5681 Leishmania peruviana

0.02 10721 1843 - 38579 Leishmania guyanensis species complex

0.01 4646 425 S 5679 Leishmania panamensis

0.01 4221 4221 - 1295824 Leishmania panamensis MHOM/COL/81/L13

0.01 4232 4232 S 5670 Leishmania guyanensis

0.05 37405 1200 - 40283 unclassified Leishmania

0.02 16009 16009 S 62297 Leishmania turanica

0.02 10578 10578 S 40284 Leishmania arabica

0.01 7782 7782 S 40285 Leishmania gerbilli

0.00 1065 1065 S 1638716 Leishmania sp. AIIMS/LM/SS/PKDL/LD-974

0.00 771 771 S 1303197 Leishmania sp. MAR LEM2494

0.02 10515 159 - 5688 lizard Leishmania

0.01 7100 7100 S 5674 Leishmania adleri

0.00 3256 3256 S 5689 Leishmania tarentolae

0.17 120110 0 G 1620386 Lotmaria

0.17 120110 120110 S 1620387 Lotmaria passim

0.06 44610 449 G 5655 Crithidia

0.04 25584 25584 S 5656 Crithidia fasciculata

0.02 13194 13194 S 59798 Crithidia acanthocephali

0.00 2183 2183 S 288676 Crithidia bombi

0.00 1708 1708 S 796354 Crithidia expoeki

0.00 1492 1492 S 796356 Crithidia mellificae

0.03 20421 0 G 5703 Endotrypanum

0.03 20421 20421 S 5705 Endotrypanum monterogeii

0.06 39861 116 - 1581334 Strigomonadinae

0.04 28491 8879 G 1003010 Strigomonas

0.02 10724 10724 S 5657 Strigomonas oncopelti

0.01 7175 7175 S 1003336 Strigomonas galati

0.00 1713 1713 S 28005 Strigomonas culicis

0.02 11254 11 G 1003337 Angomonas

0.01 10261 10261 S 59799 Angomonas deanei

0.00 982 982 S 59800 Angomonas desouzai

0.01 8418 0 G 5714 Herpetomonas

0.01 8418 8418 S 5718 Herpetomonas muscarum

0.01 6067 0 G 1470208 Paratrypanosoma

0.01 6067 6067 S 1470209 Paratrypanosoma confusum

0.00 1654 6 G 5706 Phytomonas

0.00 1014 0 S 5707 Phytomonas serpens

0.00 1014 1014 - 1276585 Phytomonas serpens 9T

0.00 482 482 S 1902498 Phytomonas francai

0.00 152 0 - 470182 unclassified Phytomonas

0.00 98 98 S 134006 Phytomonas sp. isolate EM1

0.00 54 54 S 134013 Phytomonas sp. isolate Hart1

0.00 87 0 G 1463229 Blechomonas

0.00 87 87 S 1463230 Blechomonas ayalai

**C) Results of Kraken analysis on Trypanosomatidae for the mummy FI12 (mgm_46-26489.3)**

99.80 7944391 7944391 U 0 unclassified

0.20 15736 0 - 1 root

0.20 15736 0 - 131567 cellular organisms

0.20 15736 0 D 2759 Eukaryota

0.20 15736 0 - 33682 Euglenozoa

0.20 15736 0 O 5653 Kinetoplastida

0.20 15736 846 F 5654 Trypanosomatidae

0.10 8229 73 G 5690 12Trypanosoma

0.10 8000 0 - 47570 Schizotrypanum

0.10 8000 7227 S 5693 Trypanosoma cruzi

0.00 367 367 - 1206070 Trypanosoma cruzi Tula cl2

0.00 313 313 - 366581 Trypanosoma cruzi strain Esmeraldo

0.00 51 51 - 914063 Trypanosoma cruzi JR cl. 4

0.00 38 38 - 85056 Trypanosoma cruzi marinkellei

0.00 4 4 - 1416333 Trypanosoma cruzi Dm28c

0.00 76 0 - 669453 Trypanosoma with unspecified subgenus

0.00 43 43 S 67003 Trypanosoma theileri

0.00 33 33 S 71804 Trypanosoma grayi

0.00 44 1 - 39700 Trypanozoon

0.00 39 39 S 5694 Trypanosoma equiperdum

0.00 4 0 S 5691 Trypanosoma brucei

0.00 4 0 - 5702 Trypanosoma brucei brucei

0.00 4 4 - 185431 Trypanosoma brucei brucei TREU927

0.00 29 0 - 47571 Duttonella

0.00 29 0 S 5699 Trypanosoma vivax

0.00 29 29 - 1055687 Trypanosoma vivax Y486

0.00 5 0 - 47569 Nannomonas

0.00 5 4 S 5692 Trypanosoma congolense

0.00 1 1 - 1068625 Trypanosoma congolense IL3000

0.00 2 0 - 39701 Herpetosoma

0.00 2 0 S 5698 Trypanosoma rangeli

0.00 2 2 - 429131 Trypanosoma rangeli SC58

0.08 6428 221 - 1286322 Leishmaniinae

0.05 3822 691 G 5658 Leishmania

0.02 1517 40 - 38568 Leishmania

0.01 635 0 - 38581 Leishmania major species complex

0.01 635 8 S 5664 Leishmania major

0.01 616 616 - 860569 Leishmania major strain LV39c5

0.00 7 7 - 347515 Leishmania major strain Friedlin

0.00 4 4 - 860570 Leishmania major strain SD 75.1

0.00 397 0 - 38574 Leishmania donovani species complex

0.00 397 131 S 5661 Leishmania donovani

0.00 187 187 - 1215323 Leishmania donovani Ld 2001

0.00 79 79 - 1240127 Leishmania donovani Ld 39

0.00 342 0 - 38570 Leishmania aethiopica species complex

0.00 342 0 S 5667 Leishmania aethiopica

0.00 342 342 - 1206056 Leishmania aethiopica L147

0.00 86 0 - 38582 Leishmania mexicana species complex

0.00 70 70 S 5659 Leishmania amazonensis

0.00 10 10 S 5663 Leishmania enriettii

0.00 6 0 S 5665 Leishmania mexicana

0.00 6 6 - 929439 Leishmania mexicana MHOM/GT/2001/U1103

0.00 17 0 - 38584 Leishmania tropica species complex

0.00 17 0 S 5666 Leishmania tropica

0.00 17 17 - 1206058 Leishmania tropica L590

0.01 756 30 - 40283 unclassified Leishmania

0.00 337 337 S 40284 Leishmania arabica

0.00 195 195 S 62297 Leishmania turanica

0.00 177 177 S 40285 Leishmania gerbilli

0.00 17 17 S 1638716 Leishmania sp. AIIMS/LM/SS/PKDL/LD-974

0.01 718 47 - 37616 Viannia

0.01 527 23 - 38579 Leishmania guyanensis species complex

0.00 366 366 S 5670 Leishmania guyanensis

0.00 138 0 S 5679 Leishmania panamensis

0.00 138 138 - 1295824 Leishmania panamensis MHOM/COL/81/L13

0.00 95 1 - 37617 Leishmania braziliensis species complex

0.00 86 0 S 5660 Leishmania braziliensis

0.00 62 62 - 1295825 Leishmania braziliensis MHOM/BR/75/M2903

0.00 24 24 - 420245 Leishmania braziliensis MHOM/BR/75/M2904

0.00 8 8 S 5681 Leishmania peruviana

0.00 49 0 - 38583 Leishmania naiffi species complex

0.00 49 49 S 5678 Leishmania naiffi

0.00 140 13 - 5688 lizard Leishmania

0.00 119 119 S 5674 Leishmania adleri

0.00 8 8 S 5689 Leishmania tarentolae

0.03 2001 0 G 5683 Leptomonas

0.03 1997 1968 S 5684 Leptomonas seymouri

0.00 29 29 - 1263718 Leptomonas seymouri BHU1095

0.00 4 4 S 157538 Leptomonas pyrrhocoris

0.00 297 0 G 1620386 Lotmaria

0.00 297 297 S 1620387 Lotmaria passim

0.00 44 0 G 5703 Endotrypanum

0.00 44 44 S 5705 Endotrypanum monterogeii

0.00 43 0 G 5655 Crithidia

0.00 31 31 S 796354 Crithidia expoeki

0.00 6 6 S 59798 Crithidia acanthocephali

0.00 3 3 S 5656 Crithidia fasciculata

0.00 3 3 S 288676 Crithidia bombi

0.00 193 2 - 1581334 Strigomonadinae

0.00 142 0 G 1003337 Angomonas

0.00 138 138 S 59799 Angomonas deanei

0.00 4 4 S 59800 Angomonas desouzai

0.00 49 0 G 1003010 Strigomonas

0.00 22 22 S 5657 Strigomonas oncopelti

0.00 22 22 S 1003336 Strigomonas galati

0.00 5 5 S 28005 Strigomonas culicis

0.00 16 0 G 5706 Phytomonas

0.00 11 0 S 5707 Phytomonas serpens

0.00 11 11 - 1276585 Phytomonas serpens 9T

0.00 4 4 S 1902498 Phytomonas francai

0.00 1 0 - 470182 unclassified Phytomonas

0.00 1 1 S 134006 Phytomonas sp. isolate EM1

0.00 16 0 G 5714 Herpetomonas

0.00 16 16 S 5718 Herpetomonas muscarum

0.00 8 0 G 1470208 Paratrypanosoma

0.00 8 8 S 1470209 Paratrypanosoma confusum
